# Is pulmonary artery a dose-limiting organ at risk in non-small cell lung cancer patients treated with definitive radiotherapy?

**DOI:** 10.1186/s13014-017-0772-5

**Published:** 2017-02-01

**Authors:** Jie-Tao Ma, Li Sun, Xin Sun, Zhi-Cheng Xiong, Yang Liu, Shu-Ling Zhang, Le-Tian Huang, Cheng-Bo Han

**Affiliations:** Department of Oncology, Shengjing Hospital of China Medical University, Shenyang, 110022 China

**Keywords:** Pulmonary artery, Non-small cell lung cancer, Definitive radiotherapy, Organ at risk, Survival

## Abstract

**Purpose:**

Our previous study suggested that some pulmonary artery (PA) dosimetric parameters were associated with mortality in unresectable non-small cell lung cancer (NSCLC) treated with definitive radiotherapy. The present study aims to analyze the impact of both PA and heart dosimetric parameters on survival of patients with NSCLC treated with definitive conventional fractionated radiotherapy (CFRT) in another independent research center and further determine whether the PA should be considered a dose-limiting organ at risk (OAR) for patients receiving thoracic CFRT.

**Methods:**

We performed a retrospective analysis of successive patients with medically inoperable or unresectable NSCLC treated with definitive radiotherapy or chemoradiotherapy from August 2010 to September 2014. Clinical and pathological information, PA and heart dosimetric factors, and follow-up data were collected from each patient’s records and evaluated as potential prognostic factors for survival. Survival probabilities were estimated by the Kaplan-Meier method and compared by the log rank test. Cox proportional hazards regression models were performed to determine the independent predicators of survival. The optimal cutoff points of continuous dosimetric variables were determined by Youden index in receiver operating characteristic (ROC) analysis.

**Results:**

This study analyzed the records of 141 patients, 50.4% had adenocarcinoma, 71.6% had stage III disease, and 55% patients received concurrent chemoradiotherapy. Radiation dose ranged from 60 to 76 Gy in 30–38 fractions. Median follow up was 16.9 months. Median overall survival (OS) was 20.5 months (95% confidence interval [CI] 10.3-30.7 months), and 1-, 2-, 3-year OS rates were 75.2%, 58.2% and 56%, respectively. Univariate and multivariate analysis showed that Karnofsky Performance Status (KPS) score, Charlson’s Comorbidity Index (CCI), T and N stage, PA invasion grade and the percentage of PA volume that received 40 to 55 Gy (PA V40-55) were significantly associated with OS. No significant associations were found between heart dosimetric factors and OS. Median OS of patients with PA invasion grade 0, 1, 2, and 3 were 41.8, 27.8, 12.7 and 7.5 months, respectively (*P* < 0.001). PA V40, V45, V50 and V55, using thresholds of 80%, 68%, 45%, and 32%, respectively, were independent predictors for OS.

**Conclusions:**

PA invasion grade and PA V40-55 appear associated with OS in patients with NSCLC treated with definitive CFRT. We propose that PA be considered as a dose-limiting OAR for such patients.

## Background

Lung cancer is one of the most common cancers worldwide, and non-small cell lung cancer (NSCLC) comprises 85% of lung cancer cases [1]. Radiotherapy is the primary method of treating patients with medically inoperable or unresectable locally advanced NSCLC (LA-NSCLC) [2–4]; addition of concurrent chemotherapy to radiotherapy (CRT), which is standard care for unresectable LA-NSCLC, achieves a median OS of 17–27 months [5–8]. Nevertheless, some patients suffer early recurrence and severe adverse events, such as radiation-induced pulmonary and esophageal injury, as well as massive hemoptysis.

Optimizing the radiation plan and minimizing radiation-induced toxicities requires limiting the dose to the organs at risk (OAR). Dose-volume parameters of lung, such as V20 and mean lung dose (MLD), are known to play an important role in predicting severe radiation pneumonitis and early deaths [9]. The pulmonary artery (PA) is the most commonly tumor-involved thoracic great vessel [10, 11]. Patients who had PA invasion by tumor were usually considered to have unresectable disease, though vascular reconstruction has been applied in selected patients. Our previous study of 100 patients with inoperable NSCLC in the United State first categorized the grade of PA invasion by contrast-enhance Computed Tomography (CT) or Positron Emission Tomography (PET) scan and indicated that only 2 out of 4 patients with grade 5 PA invasion experienced massive bleeding caused death, and overall grades of PA invasion were not associated with overall survival in patients with inoperable NSCLC. However, in patients with LA-NSCLC treated with concurrent chemoradiation, the proportion of PA volume that received more than 45–60 Gy was associated with shorter OS [12].

The heart is known to be an important OAR for patients receiving thoracic radiotherapy. Postoperative radiation therapy for lung cancer was associated with increased mortality from heart disease [13]. In the RTOG 0617 trial, there were less than 5% cardiac adverse events (≥ grade 3) in either the high dose or low dose arm, and multivariate analysis showed that the higher proportions of heart volume receiving ≥ 5 Gy (H V5) or ≥ 30 Gy (H V30) were significantly correlated with shorter survival [7]. However, Tucker et al. [14] did not confirm the result of that trial regarding the impact of heart dose volume on OS.

In our previous study [12], the heart was not evaluated in the univariate or multivariate analysis. In addition, data from another independent research center is needed to confirm these findings regarding the PA. Hence, we performed a retrospective analysis to re-evaluate the results of the previous study and verify the impact of dosimetric parameters of the PA and heart on the survival of patients with NSCLC treated with definitive conventional fractionated radiotherapy (CFRT) with or without chemotherapy and to determine whether PA should be considered an OAR and what PA dose/volume limit should recommended for patients receiving definitive CFRT.

## Methods

### Patients

We retrospectively reviewed the records of successive patients with pathologically confirmed and medically inoperable or unresectable stage I-IIIB NSCLC at Shengjing Hospital between August 2010 and September 2014. Patients who had good Karnofsky Performance Status (KPS) (≥70), acceptable forced expiratory volume in 1 s (FEV1) (>1.2 L) or FEV1% (>70%), and received the definitive conventionally fractionated radiotherapy with or without concurrent chemotherapy were included in this study. Comorbidities, including hypertension, cardiac-cerebral vascular disease, and chronic obstructive pulmonary disease, were calculated by Charlson’s Comorbidity Index (CCI) [15, 16]. This study was approved by the local ethics committee of our hospital. All patients provided written informed consent prior to participating in the study.

### Radiotherapy parameter extraction

Radiotherapy was carried out using intensity modulated radiation therapy (IMRT) or 3-dimensional conformal radiatherapy (3DCRT) techniques. Targets contours were defined as follows: gross tumor volume (GTV) included primary lesion (GTVp) and metastatic lymph nodes (GTVnd), which were either pathologically confirmed, had a short diameter ≥ 1 cm, or were PET positive; clinical target volume (CTV) was defined by GTVp plus a 6–8 mm margin and the involved lymph node region; planning target volume (PTV) was defined by CTV plus a 0.5 cm margin for axial direction and a 1–1.5 cm margin for head-foot direction. A total dose of 60–76 Gy in 30–38 fractions was delivered with 2.0 Gy daily fractions over 6–7.5 weeks using 6 MV photons. The prescribed dose covered 95% of the PTV. The dose limits for the organs at risk (OAR) were as follows: bilateral lung: V20 (percentage of volume receiving more than 20 Gy) ≤ 30% and mean lung dose (MLD) ≤ 20 Gy; spinal cord: maximum dose of planning organ at risk volume (PRV) ≤ 48–52 Gy; heart: V30 ≤ 40-60% and V40 ≤ 30-50%; esophagus: V50 ≤ 50%. The PA and heart were contoured according to Radiation Therapy Oncology Group (RTOG) 1106 atlas. Dosimetric parameters were extracted from the treatment planning system (Oncentra, Elekta company, Sweden), including mean and maximum doses to the heart or PA as well as the percentage of PA or heart volume that received a specific dose (from V5, 10, 15 to V70 every 5th interval). The evaluation of the grades of PA invasion included primary tumor and metastatic lymph nodes invading PA. PA invasion grade criteria were slightly modified from those described previously [12] and are summarized in Table 1. As per these modified criteria, PA invasion was graded as 0, 1, 2, and 3 for no, minimal, moderate, and extensive invasion, respectively.

**Table 1:** Grading criteria of pulmonary artery invasion^a^

Grade	Definition based on CT contrast
Grade 0 (no invasion)	No evidence of vessel invasion, ≥1 mm from the closest pulmonary vessel wall (presence of a fat plane between tumor and vessel wall)
Grade 1 (minimal invasion)	Tumor invasion with 0 mm to the closest pulmonary vessel wall, no fat plane, without presence of narrowing or truncation of vessels, nor signs of vessel wall damage (irregularity, discontinuity or intra-luminal mass formation)
Grade 2 (moderate invasion)	Circumferential involvement with narrowing or truncation
Grade 3 (extensive invasion)	Tumor invading pulmonary vessel extensively with any sign of vessel wall damage: irregularity, discontinuity or intra-luminal mass formation or massive haemorrhage due to the tumor invading pulmonary artery

^a^Revised from [12]

### Follow-up

All patients underwent follow up with chest CT scan every 3 months during the first two years after radiotherapy and every 6 months thereafter. In addition, abdominal CT, bone Emission Computed Tomography and brain Magnetic Resonance Imaging scans were performed every 6 months or as clinically indicated. OS was calculated from the start of radiotherapy until any cause of death or the last date of follow-up. Patients still alive at the last follow-up date (Sep 25, 2015) were censored on that day, and loss to follow-up was considered as a censored event. Progression-free survival (PFS) was calculated from the start of radiotherapy until the first imaging diagnosis of recurrent or progressive disease. Local progression-free survival (LPFS) was based on recurrence within the radiation field.

### Statistical analyses

The potential prognostic factors for univariate analyses of survival included patients characteristics, such as age; gender; score of KPS, CCI; tumor associated factors, such as tumor location, clinical stage, tumor (T) stage, lymph nodal (N) stage, histologic type, and PA invasion grade; treatment associated factors, such as treatment modality (radiotherapy alone or sequential CRT or concurrent CRT), radiotherapy technique (IMRT or 3DCRT), and RT dose; as well as dosimetric parameters, such as minimum dose for 95% volume of GTV, CTV and PTV (D95), the percentage of PA volume (PA V5, 10, 15, and 20–70) and heart volume (H V5, 10, and 15–70) that received a specific radiation dose (Table 2). Pearson correlation coefficient was used to examine the correlation between potential prognostic factors. Survival was estimated using the Kaplan-Meier method and curves were compared using the log rank test. Both univariate and multivariate analyses were performed to evaluate associations between potential prognostic factors and OS. Multivariable Cox proportional hazard models were used to calculate adjusted hazard ratios (HRs) and their 95% confidence intervals (CIs); variables likely to be associated with OS, based on *P* values of less than 0.1 from the univariate analysis, were included in these models. Simultaneous inclusion in the same multiple variable regression model of two highly correlated variables (correlation co-efficient greater than 0.7) was considered inappropriate. Consequently, for multivariable analysis, such variables were entered separately, one by one, with other independent variables. The optimal cutoff points of significant continuous variables were determined by Youden index in receiver operating characteristic (ROC) analysis. Area under the curve (AUC) determined by ROC analysis was used to estimate the predictive ability of covariates for survival status; cutoff values were evaluated and confirmed by repeated Kaplan-Meier and multivariate Cox analyses. The software of IBM SPSS version 21.0 was used for statistical analysis, a *P* value less than 0.05 was considered statistically significant.

**Table 2:** Clinicopathologic characteristic

Characteristic	Number (%)	Characteristic	Number (%)
Age (years)		RT modality	
Median (range)	60 (39–85)	RT alone	55 (39%)
< 60	72 (5.1)	concurrent CRT	47 (33.3%)
≥ 60	69 (48.9)	sequential CRT	39 (27.7%)
Gender		Tumor stage	
Male	97 (68.8%)	T1	26 (18.4%)
Female	44 (31.2%)	T2	46 (32.6%)
KPS (70–100)		T3	38 (27%)
70–80	5 (3.5%)	T4	31 (22%)
80–90	28 (19.9%)	Lymph nodes stage	
90–100	108 (76.6)	N0	42(29.8%)
Smoking		N1	48 (34%)
No	68 (48.2%)	N2	32 (22.7%)
Yes	73 (51.8%)	N3	19 (13.5%)
Weight loss (%)		Clinical stage	
Median (range)	3 (0–10)	I	5 (3.5%)
≤ 3%	72 (51.1)	II	47 (33.3%)
> 3%-10%	69 (48.9)	IIIA	62 (44%)
COPD		IIIB	27 (19.1%)
No	130 (92.2%)	PA invasion grade	
Yes	11 (7.8%)	Grade 0	49(34.8%)
CVD		Grade 1	36(25.5%)
No	108 (76.6%)	Grade 2	37(26.2%)
Yes	33 (23.4%)	Grade 3	19(13.5%)
Hypertension		Target volume (cc) median (range)	
No	109 (77.3%)		
Yes	32 (22.7%)	GTV	43.6 (9–400.9)
CCI		CTV	92.2 (12.56–843.6)
Median (range)	3 (0–7)	PTV	213.7 (23.5–1025.5)
< 3	62 (44.0%)	D95 to targets (Gy) median (range)	
≥ 3	79 (56.0%)		
Tumor location		D95 to GTV	64.9 (48.1–74.0)
Central	116 (82.3%)	D95 to CTV	63.1 (46.4–71.9)
Peripheral	25 (17.7%)	D95 to PTV	60.1 (45.6–69.9)
Pathology		D_max_ to PA (Gy)	
Adenocarcinoma	71 (50.3%)	Median (range)	65.8 (0.6–76.2)
Squamous	61 (43.3%)	D_mean_ to PA (Gy)	
Large-cell & NOS	9 (6.4%)	Median (range)	37.9 (0–71.3)
RT technique		D_max_ to Heart (Gy)	
IMRT	52 (36.9%)	Median (range)	56 (0.2–65.7)
3DCRT	89 (63.1%)	D_mean_ to Heart (Gy)	
RT dose (Gy)		Median (range)	5.2 (0–34.9)
Median (range)	66 (60–76)		
< 66	62 (44.0%)		
≥ 66	79 (56.0%)		

*KPS* Karnofsky performance status, *COPD* chronic obstructive pulmonary disease, *CVD* Cardiac vascular disease, *CCI* Charlson comorbidity index, *NOS* NSCLC not otherwise specified, *IMRT* intensity modulated radiation therapy, *3DCRT* three-dimensional conformal radiation therapy, *RT* radiotherapy, *Chemo* Chemotherapy, *GTV* gross tumor volume, *CTV* clinical target volume, *PTV* planning target volume, *D95* minimum dose to 95% volume of targets, *PA* pulmonary artery, *cc* cubic centimeter, *Gy* gray, *Dmax* Maximum dose, *Dmean* Mean dose

## Results

### Characteristics of patients

One hundred forty-one patients with pathologically confirmed stage I-IIIB NSCLC were eligible for this study. Patient characteristics are summarized in Table 2.

### Correlation of potential prognostic factors

The correlations among clinicopathologic factors and dosimetric parameters were calculated. RT dose was not correlated with GTV, CTV and PTV; Pearson correlation coefficients were 0.171, 0.121 and 0.064, respectively, all *P* values were > 0.05. Both T stage and N stage were not correlated with volumes of GTV, CTV or PTV or D95 to target volumes, Pearson correlation coefficients ranged from −0.126 to −0.291, all *P* values were > 0.05. In addition, no correlations were noted between heart dosimetric parameters and target volumes (P > 0.05). Only weak correlations were found between target volumes and some PA dosimetric parameters (PA V35-60) and between H V40-65 and PA V35-65, but all Pearson correlation coefficients were less than 0.4, *P* < 0.05.

#### Results of univariate survival analysis

**Fig. 1:**
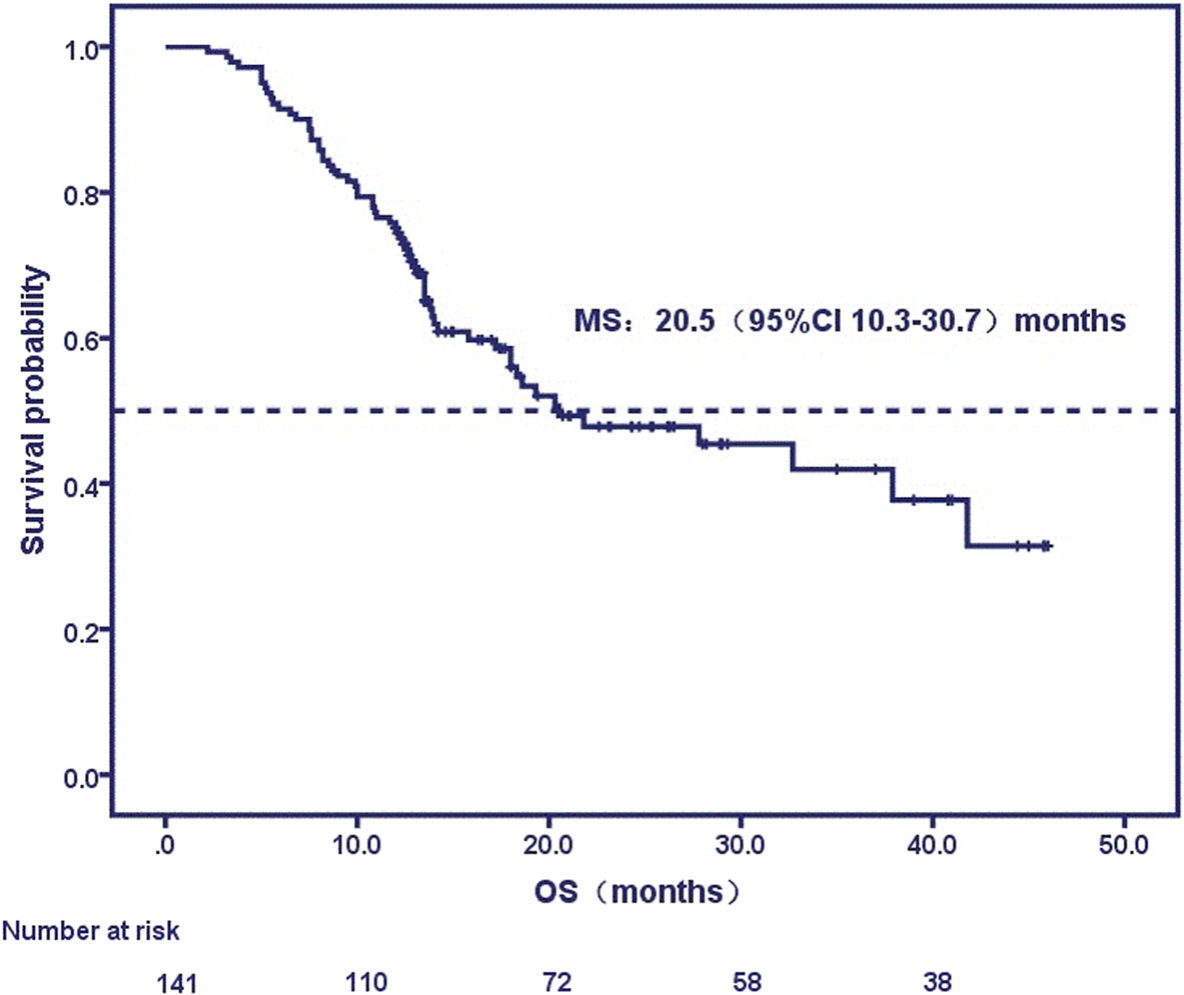
Curve of overall survival (OS)

The median follow-up for the entire cohort was 16.9 months (range: 6.5 to 42.0 months). Median PFS, LPFS, and OS were 8.4 months, 12.4 months, and 20.5 months (95% CI: 9.58-31.42 months), respectively, and the 1-, 2- and 3-year OS rates were 75.9%, 58.2% and 56%, respectively (Fig. 1). Univariate analysis showed that age, gender, status of smoking, weight loss, tumor location, treatment modality, RT dose, D95 to target volumes, maximum and mean dose to the PA or heart were not related with OS. However, KPS score, T stage, N stage, clinical stage, CCI, PA invasion grade, GTV, CTV, PTV, PA V20, 25–65 and H V30, 35–45 were significantly associated with median OS (*P* < 0.1) (Table 3).

**Table 3:** The result of multivariate analysis of significative potential prognostic factors associated with overall survival by using univariate analysis

	Univariate		Multivariate		
	Variables	*P* value	*P* value	HR	95% CI
Categorical Variables	CCI	0.00001	0.00001	1.816	1.386–2.378
	T-stage	0.0004	0.0001	3.160	1.944–5.138
	N-stage	0.007	0.001	1.907	1.315–2.767
	Clinical stage	0.001	0.493	0.697	0.248–1.96
	PA invasion grade	0.028	0.0001	2.172	1.467–3.216
Continuous Variables	KPS	0.001	0.043	0.934	0.886–0.936
	GTV	0.015	0.066	0.986	0.975–0.997
	CTV	0.045	0.07	1.01	1.003–1.017
	PTV	0.081	0.203	0.998	0.995–1.001
	PA V10	0.099	0.698	0.275	0–16.913
	PA V15	0.098	0.792	0.309	0–15.602
	PA V20	0.084	0.678	1.691	0–4.357
	PA V25	0.094	0.468	0.022	0–6.387
	PA V30	0.002	0.553	8.474	0.007–9.855
	PA V35	0.001	0.111	2.367	0.271–2.967
	PA V40	0.001	0.043	2.113	1.014–4.936
	PA V45	0.0004	0.0001	2.660	1.089–5.717
	PA V50	0.001	0.0001	1.203	0.062–2.056
	PA V55	0.002	0.05	1.489	0.098–2.096
	PA V60	0.005	0.191	0.076	0.002–3.621
	PA V65	0.027	0.149	0.069	0.002–2.598
	H V30	0.062	0.148	0	0–4.075
	H V35	0.059	0.227	1.725	0–9.681
	H V40	0.035	0.755	0.001	0–5.625
	H V45	0.092	0.923	5.765	0–12.916

*CCI* Charlson comorbidity index, *KPS* karnofsky performance status, *GTV* gross tumor volume, *CTV* clinical target volume, *PTV* planning target volume, *D95* minimum dose to 95% volume of targets, *PA* pulmonary artery, PA V5, 10–70: the percentage of PA volume receiving 5,10 to 70Gy, *PA*_*max*_ the maximum dose to PA, *PA*_*mean*_ the mean dose to PA, H V5,10-75: the percentage of heart volume receiving 5, 10 to 70Gy, *H*_*max*_ the maximum dose to heart, *H*_*mean*_ the mean dose to heart

#### Results of multivariate survival analysis

**Fig. 2:**
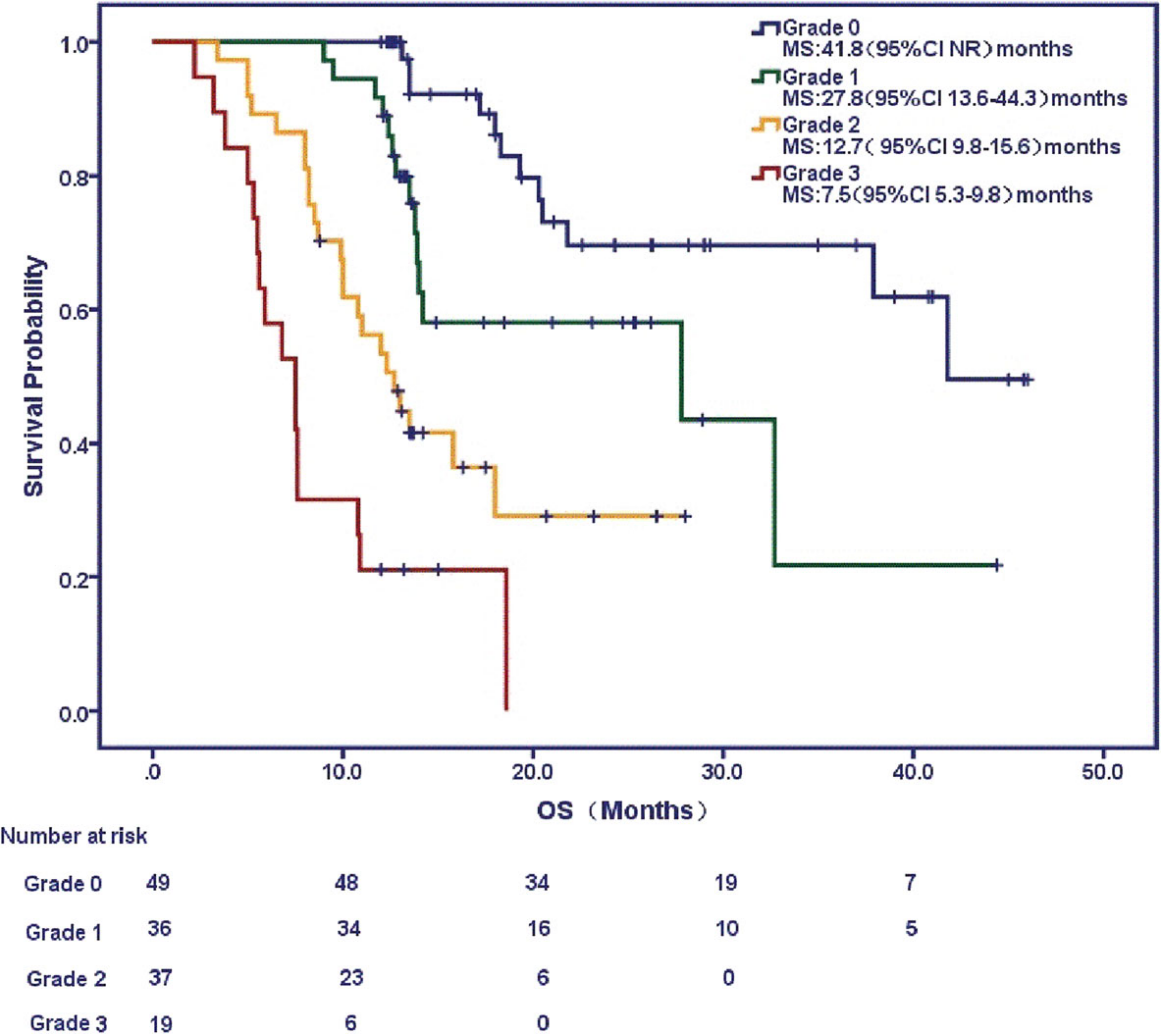
Curve of overall survival (OS) by pulmonary artery (PA) invasion grade: grade 1 (no invasion), grade 2 (minimal invasion), grade 3 (moderate invasion), and grade 4 (extensive invasion)

Multivariate analysis indicated that the clinicopathologic factors significantly associated with shorter OS were lower KPS score (< 85), higher CCI (≥ 3), higher T (T3, T4) stage, N (N2, N3) stage, and higher grade of PA invasion. Median OS was 13.1 months vs. 32.7 months in patients with KPS < 85 vs. ≥ 85, respectively; was 10.9 months vs. 37.9 months in CCI ≥ 3 vs. < 3, respectively; was 13.5 months vs. 41.8 months in T3, 4 stage vs. T1, 2 stage, respectively; and was 19.3 months vs. 27.8 months in N2, 3 stage vs. N0, 1 stage, respectively. All *P* values were less than 0.05. In addition, the median OS and 2-year survival rates were 41.8 months and 79.6%, 27.8 months and 66.67%, 12.7 months and 37.84%, and 7.5 months and zero, respectively, for no invasion (grade 0), minimal (grade 1), moderate (grade 2), and extensive invasion (grade 3), *P* < 0.05 (Fig. 2 and table 4). Two patients with grade 3 PA invasion (2/19, 10.5%) died of massive bleeding after radiotherapy, and no other bleeding events or rupture of great vessel were observed during or after radiotherapy.

**Table 4:** Overall survival (OS) of different grade of PA invasion

Grade	n	Median OS (95%CI) (months)^*^	1-year OS rate (%)	2-year OS rate (%)
0	49	41.8 (−)	100	79.6
1	36	27.8 (19.96–32.36)	91.67	66.67
2	37	12.7 (9.85–15.56)	54.05	37.84
3	19	7.5 (5.25–9.75)	21.05	0

*P = 0.0003 for trend

**Fig. 3:**
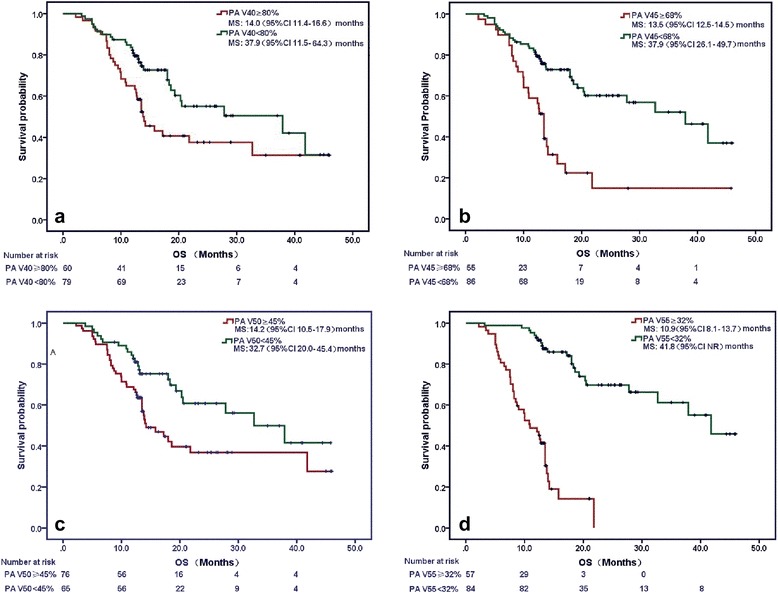
Survival curves by the percentage of PA volume that received 40-55Gy. (**a**) PA V40 ≥ 86% vs. < 86%; (**b**) PA V45 ≥ 68% vs. < 68%; (**c**) PA V50 ≥ 45% vs. < 45% and (**d**) PA V55 ≥ 32% vs. < 32%

**Fig. 4:**
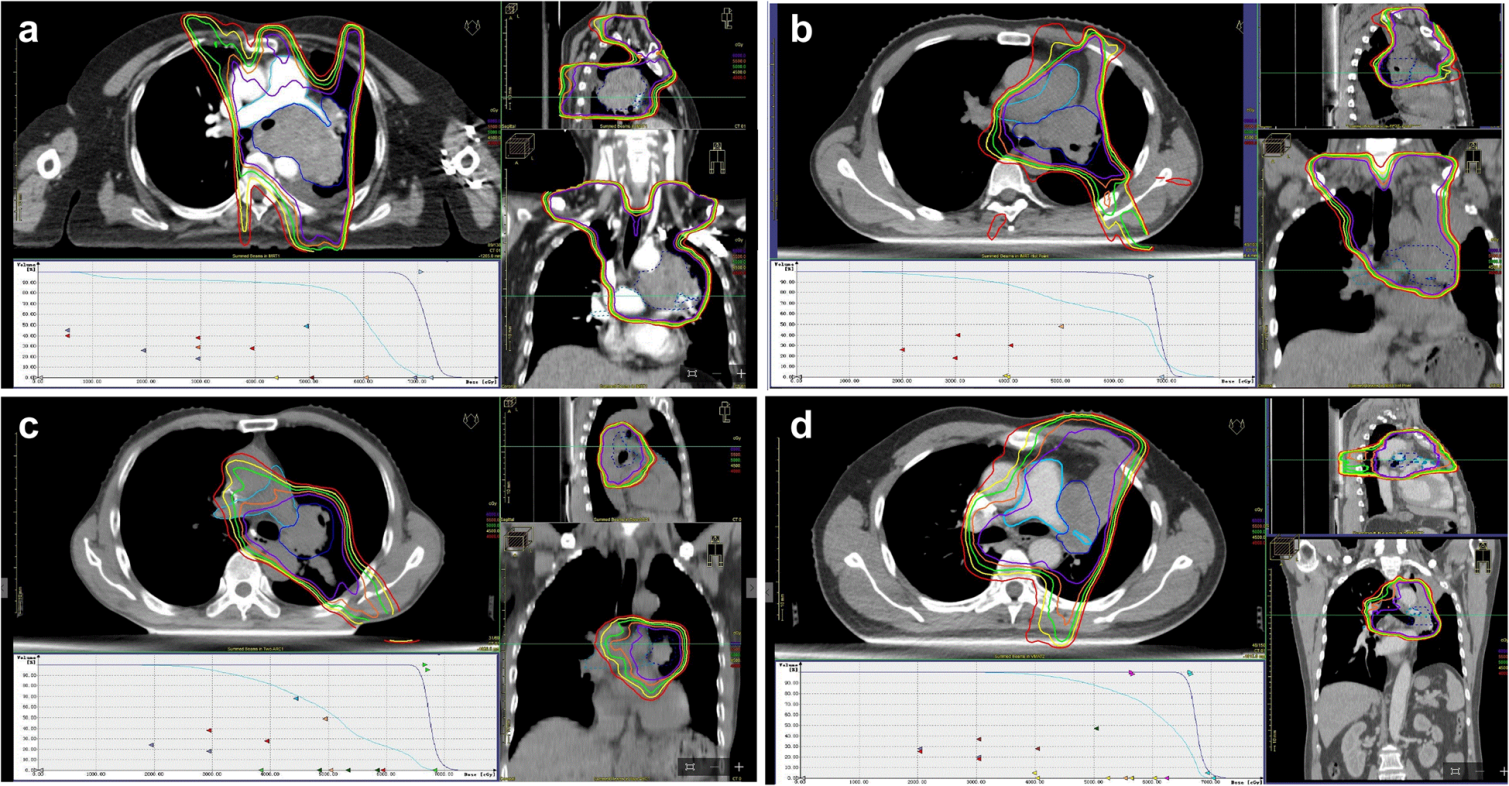
Four patients with high grade of pulmonary artery (PA) invasion and high PA V40-55 values had short overall survival (OS). Dose volume histograms (DVH) of PA (*azure*) and PTV (*blue*), as well as isodose curves of 40 Gy (*red*), 45 Gy (*yellow*), 50 Gy (*green*), 55 Gy (*orange*), and 60 Gy (*purple*) in axial, sagittal, and coronal views are shown. PA V40-60 was the percentage of PA volume of PA that received 40-60 Gy. **a** A patient who was diagnosed with non-small cell lung cancer (NSCLC) (T4N3M0 and PA invasion grade 3) died of massive bleeding approximately 6 months after radiotherapy. PA V40, V45, V50, V55, and V60 were 90%, 89.02%, 86.89%, 81.03%, and 56.81%, respectively. **b** A patient who was diagnosed with NSCLC (T2N2M0 and PA invasion grade 1) survived 9.9 months after radiotherapy. PA V40, V45, V50, V55, and V60 were 87.46%, 80.09%, 72.36%, 66.31%, and 61.19%, respectively. **c** A patient who was diagnosed with NSCLC (T3N0M0 and PA invasion grade 2) survived 8.7 months after radiotherapy. PA V40, V45, V50, V55, and V60 were 81.28%, 69.78%, 55.03%, 33.2%, and 22.72%, respectively. **d** A Patient who was diagnosed with NSCLC (T4N1M0 and PA invasion grade 3) survived 5.5 months after radiotherapy. PA V40, V45, V50, V55, and V60 were 97.81%, 94.12%, 87.83%, 79.45%, and 64.06%, respectively

With regard to dosimetric factors, multivariate analysis demonstrated that volumes of targets including GTV, CTV, PTV and the percentages of heart volume that received a specific radiation dose, were not significantly associated with OS. However, PA V40-55 Gy were significantly associated with OS independent of KPS and stage. According to the ROC curves, the best cutoff values for PA V40, 45, 50 and 55 predictive of OS were 80%, 68%, 45%, and 32%, respectively. Median OS rates were 14 months vs. 27.8 months, respectively in patients with PA V40 ≥ 86% vs. < 86%, *P* < 0.0001; were 13.5 months vs. 37.9 months, respectively in patients with PA V45 ≥ 68% vs. < 68%, *P* < 0.0001; were 14.2 months vs. 32.7 months, respectively in patients with PA V50 ≥ 45% vs. < 45%, *P* < 0.0001; and were 10.9 months vs. 41.8 months, respectively in patients with PA V55 ≥ 32% vs. < 32%, *P* < 0.0001 (Fig. 3a-d). The dose volume histograms (DVH) and dose-curves of four patients presenting with high grades of PA invasion and PA V40-55 are shown in Fig. 4a-d.

## Discussion

The present study demonstrated that the score of KPS, CCI, stages of T and N, PA invasion grade, as well as percentage of PA volumes that received 40–55 Gy were independent prognostic factors of OS for patients with NSCLC treated with definitive CFRT. PA invasion grade was significantly associated with OS, though it was not associated with bleeding during or after radiotherapy. In addition, patients with PA V40 > 80%, V45 > 68%, V50 > 45% or V55 > 32% had a significantly shorter OS. The results further confirmed our previous conclusions [12] and suggested that the PA should be regarded as an OAR during conventionally fractionated thoracic radiotherapy or chemoradiotherapy, and that the grade of PA invasion and PA dosimetric parameters V40-55 could be used to predict survival for those patients with NSCLC who received definitive CFRT.

Radiation induced lung injury, esophagitis, and heart injuries are the most frequent radiation related toxicities and have been widely investigated recently. The RTOG 0617 trial [7] revealed that high dose radiotherapy given to patients with LA-NSCLC failed to improve local control and prolong OS compared with standard dose radiotherapy. The possible damage to the local immune microenvironment of the field receiving high dose radiation, and the radiation related toxicities might be the major reason why patients with high dose radiotherapy had shorter survival. In this trial, H V5 and H V30, but not radiation modalities were correlated with OS [7]; The present study also evaluated the relationship between OS and heart dosimetric parameters including maximum or mean dose to the heart, or the percentage of heart volume that received a specific dose (H V5-70). However, no significant associations were found between them, although the radiation related heart injury was not evaluated herein. The reason for this finding might be attributed to relatively strict radiation dose constraints for heart that were used in our study. Our results were in accordance with Tucker et al’s study [14], which found that heart dosimetric parameters did not significantly affect survival.

A great vessel is deemed as a dose-limiting organ when lesions are given hypofractionated or stereotactic body radiotherapy (SBRT) [17]. Some researchers thought there were greater risks of severe hemoptysis and early mortality among patients who had severe PA invasion during or after radiotherapy. A previous study stated that vessel rupture may result from the penetration of the tumor into the vessel wall itself [18]. However, a later study [19] showed that this explanation might be implausible because penetration of a tumor into the wall of an elastic artery is so unusual except in the following circumstances: the vessel itself (e.g., atherosclerosis), had exposed vasa vasorum during surgery and damage to the adventitia caused by ulcer, fistulas or infection around artery that were independent of radiotherapy [19]. Another study showed that toxicity to the aorta after reirradiation was relatively rare, even when maximum composite doses to the aorta exceeded 100 Gy [20].

Recently, our study [12] first proposed grading criteria for PA invasion from grade 0 to grade 5, describing the distance of tumor to the PA, degree of circumferential involvement of the PA, and status of vessel wall damage. In that study, we found patients with PA invasion of grade 0–1 had a longer median OS than those with grade 2–5 (33.4 months vs. 18.3 months, *P* = 0.242), and two of the 4 patients with grade 5 PA invasion died suddenly from massive hemorrhage at 3 and 4.5 months after completion of radiotherapy. To facilitate its clinical use, we simplified the grading system to grade 0, 1, 2, 3 for no, minimal, moderate, and extensive invasion, respectively. In this group of patients, we found a significant relationship between grade of PA invasion and survival. Two patients with extensive PA invasion died of massive bleeding after radiotherapy. We believe that a higher grade of PA invasion indicates worse clinicopathologic behavior and prognosis, though few bleeding events were observed during or after radiotherapy in both studies. In the future, reliable imaging or pathological assessment of great vessel rupture needs to be developed to confirm this assumption.

The present study also found that the percentages of PA volume that received 40–55 Gy were significantly associated with OS regardless of target volume, KPS, CCI and stage. Especially, patients with PA V45 > 68%, and those with PA V55 > 32% had a significantly shorter OS. Interestingly, the results were highly consistent with our previous study in which high dose volume of PA V45-60 was predictive of shorter OS, and the cut-off values of PA V45 and V60 were 70% and 37%, respectively. Both studies suggest that PA V45 (68-70%) is a better cut-off value predictive of shorter OS in patients with LA-NSCLC treated with CFRT. Our findings differed from those using SBRT, in which maximum dose exceeding 50 Gy to the PA was related with massive hemoptysis and caused death [21]; in our study, when CFRT was administrated, the maximum dose to the PA was not related with OS.

In the present study, we found that GTV, CTV, PTV and dose coverage of target volumes were not associated with OS, which seemed contradictory to the effect of the factors T or N stage on OS, because a higher T or N stage often results in a larger target volume. However, we did not see any correlation between T or N stage and the target volumes GTV, CTV or PTV. Correlation analysis revealed only weak correlations between target volumes and PA V40-V55, thus ruling out an influence of target volume on PA volume dose. Certainly, our study may be too small to distinguish the independent or synthetic effect of heart and PA dosimetric parameters on survival. Future prospective studies with larger numbers of patients are needed to pursue the combined effects of heart and PA dosimetry. In addition, in our study only two patients with extensive PA invasion died of fatal hemoptysis after radiotherapy during follow-up. Hence, it appears that in this cohort, these rare outcomes, such as fatal hemoptysis or massive bleeding, are not good end points guide recommendations for dosimetric constraints for this OAR. PA grading variation from the start of radiotherapy to death may be an alternative end point. However, this is not evaluated in the present study because the accurate PA grading variation largely depends on reliable imaging or pathological analysis of abnormalities, such as ulcer, fistula, and stricture. On the other hand, we believe that the PA is an OAR similar to the heart, and that higher dose-volume irradiation to the PA might also cause serious consequences such as pericarditis, arrhythmia, and fatal hemoptysis. Future studies are needed to validate these postulations.

## Conclusion

PA invasion grade and PA V40-55 were independent factors predictive of OS regardless of KPS, CCI and stage in patients with NSCLC treated with definitive CFRT. We proposed that the PA should be regarded as an organ at risk relevant to target delineation and dose limitation during CFRT. Limiting moderate-to-high PA dose volume, especially PA V45, is recommended due to its better ability to predict survival.

## Abbreviations

3DCRT: 3-Dimensional conformal radiotherapy
AUC: Area under the curve
CCI: Charlson’s comorbidity index
CFRT: Conventional fractionated radiotherapy
COPD: Chronic obstructive pulmonary disease
CRT: Concurrent chemotherapy to radiotherapy
CT: Computed tomography
CTV: Clinical target volume
CVD: Cardiac vascular disease
FEV1: Forced expiratory volume in 1 s
GTV: Gross tumor volume
IMRT: Intensity modulated radiation therapy
KPS: Karnofsky performance status
LA-NSCLC: Locally advanced non-small cell lung cancer
LPFS: Local progression-free survival
MLD: Mean lung dose
NSCLC: Non-small cell lung cancer
OAR: Organ at risk
OS: Overall survival
PA: Pulmonary artery
PET: Positron emission tomography
PFS: Progression-free survival
PRV: Planning organ at risk volume
PTV: Planning target volume
ROC: Receiver operating characteristic
RTOG: Radiation therapy oncology group
SBRT: Stereotactic body radiotherapy
